# No Prescription, No Problem! A Mixed-Methods Study of Antimicrobial Stewardship Relating to Working Equines in Drug Retail Outlets of Northern India

**DOI:** 10.3390/antibiotics9060295

**Published:** 2020-06-02

**Authors:** Caroline Nye, Tamlin Watson, Laura Kubasiewicz, Zoe Raw, Faith Burden

**Affiliations:** 1 Centre for Rural Policy Research (CRPR), The University of Exeter, EX4 4PJ Devon, UK; 2 The Donkey Sanctuary, Sidmouth, EX10 0NU Devon, UK; tamlin.watson@thedonkeysanctuary.org.uk (T.W.); laura.kubasiewicz@thedonkeysanctuary.org.uk (L.K.); zoe.raw@thedonkeysanctuary.org.uk (Z.R.); faith.burden@thedonkeysanctuary.org.uk (F.B.)

**Keywords:** antimicrobial resistance, social drivers, LMICs, antimicrobial stewardship, India, community pharmacy, antibiotic dispensing

## Abstract

Multidrug resistance (MDR) is already occurring among some equids in India. Donkeys and mules are a mobile species moving between regions and international borders, often populating areas of India where private community pharmacies, or medical stores, are the primary healthcare provider for both humans and animals. This article highlights how the capacities of drug retail outlet workers might affect their antibiotic dispensing practices, particularly in relation to donkeys and mules, in order to consider how this might impact the development of antimicrobial resistance (AMR) on a wider scale. A mixed-methods approach was implemented using patient simulation method (*n* = 28), semi-structured interviews (SSIs) (*n* = 23), focus group discussions (FGDs) with veterinary practitioners and non-governmental organisation animal health workers (*n* = 2 FGDs), and participant observation. Fewer than 48 per cent of drug retail outlet workers admitted to having had any formal training in pharmaceuticals at all, while 78 per cent reported having no formal training in animal-related pharmaceuticals. Moreover, 35 per cent of all participants sold antibiotics without a prescription, unprompted and without specifically being asked for antibiotics. Of the antibiotics dispensed, only 21 per cent were correctly dispensed for the symptoms presented, and all dosages dispensed were incorrect (underdosed). Furthermore, 43 per cent of drug retail outlet workers interviewed believe that some antibiotics can be legally dispensed without a prescription. Equine owners in northern India are frequently being sold antibiotics without a prescription and, in most cases, with incorrect diagnoses, treatment choice, and dosage. A substantial gap in capacities exists amongst Drug Retail Outlet (DRO) workers, with few being sufficiently qualified or trained to dispense antibiotics to animal owners. The study highlights the need for further training of private DRO workers as well as knowledge extension and awareness training for both DRO workers and animal owners regarding antimicrobial resistance and its potential impact upon livelihoods. It also illustrates the need to identify a balance whereby greater enforcement of regulation at all levels is implemented, while at the same time maintaining sufficient access to medicine for rural populations.

## 1. Introduction

### 1.1. Role of Antibiotics

Antimicrobial drugs play a pivotal role in human and animal healthcare, yet developing resistances to these drugs pose a very significant threat to the future of their effectiveness, portending a potential global crisis in disease management. Such mechanisms of resistance can result from natural occurrences in the environment, but the primary cause accelerating the problem of antimicrobial resistance is poor stewardship of the drugs themselves [1]. While the pharmaceutical industry has decelerated its development of new antimicrobial drugs, meaning that any new antibiotics might take up to ten years to reach the market, emphasis now rests on maintaining the effectiveness of current drugs for as long as possible [2]. In low- and middle-income countries (LMICs), however, where antimicrobial stewardship is most poorly developed, a critical dichotomy is in play. Any attempt to understand disease burden and management must consider both patient access to life-saving drugs, and the effect of policy regulation enforcement on antibiotic availability, as ‘lack of access to effective and affordable antibiotics still kills more children in India than does drug resistance’ [3] (p. 4).

### 1.2. Antimicrobial Stewardship

Complacency, misuse, and overuse of antibiotics, are recognised as significant contributing factors to the growing global issue of antimicrobial resistance. This is prevalent in veterinary health and livestock management as well as human healthcare [4]. The term ‘antimicrobial stewardship’ has largely been associated with the prescribing practices of practitioners, such as doctors, veterinarians and, in many countries where enforcement of dispensing regulations are limited, pharmacists. Dyar et al., [5] however, suggest that the term antimicrobial stewardship should be defined as ‘a coherent set of actions which promote using antimicrobials responsibly’ [5] (p. 796). This extends the role of responsibility beyond simply that of the prescriber or dispenser to any other stakeholder capable of exercising responsibility towards antimicrobial usage. To date, the majority of studies regarding poor stewardship have focussed on the prescribing practices of doctors due to their role as ‘key gatekeepers for medication access’ [6] (p. 2). Studies examining the role of pharmacies’ contribution to antimicrobial resistance have increased since 2000, yet they are still considered ‘lacking’ [6] (p. 2). This is especially the case regarding the stewardship role of providers in veterinary medicine [7]. The majority of studies generally tend to employ simulated patient scenarios only [8,9,10] while more comprehensive studies examining both their dispensing practices and educational experiences are absent [6]. This is especially the case, again, with regards to animal-related prescribing [11].

### 1.3. Antimicrobial Resistance and Equines

While some literature exists regarding the use of antibiotics by, and prescribing patterns for, dairy, poultry and other livestock sectors [11,12], no research has so far examined that dispensed to equine owners and more specifically, donkey and mule owners. With a global population of over 110 million equines, 48 per cent of which are donkeys or mules used primarily as working animals and therefore of considerable importance to many local communities, disease awareness of these species as a whole has been limited [13], especially with regards to antimicrobial resistance and its related drivers. Where equine disease is considered in research literature, studies tend to focus on horses as opposed to donkeys or mules, as they are associated with higher value pursuits, such as entertainment and competition. Meanwhile working animals or ‘beasts of burden’ [14] (p. 1121), such as mules and donkeys, are often perceived as being of lower value due to their not being directly production-related. Linking practices regarding prescribing and dispensing of medicines for donkeys and mules has, as a consequence, been completely ignored [13]. 

As research regarding the transfer and spread of antimicrobial resistance (AMR) in the environment develops, species that are highly mobile within the environment require further examination. Faecal shedding of bacteria into the environment is known to occur in domestic animals, for example, Burkholderia pseudomallei from equids [15]. This coupled with a propensity for inter-and intra- species gene exchanges, particularly from commensal and environmental bacteria to pathogenic species, has considerable ramifications for human health [16]. Translocation of equids over long distances into areas where human inhabitants may be immunocompromised or where access to health services is sometimes limited has obvious implications for disease and the dissemination of AMR. While certain livestock species, such as buffalo, sheep, and goats, roam within a limited boundary in LMICs, such as India and Nepal, donkeys and mules tend to be transported across larger distances due to the seasonal and mobile nature of the work they are attached to, usually brick kilns or construction sites. The purpose of some mules can also transmute frequently between cargo of bricks or materials to that of humans at pilgrimage sites, requiring travel across great distances, including between countries [17]. Equine markets where working equids often congregate also act as a potential hub where ‘diseases can easily be transmitted from one to another’ [17] (p. 99). Such movement could therefore (like wildlife) have ‘the potential to move AMR from hotspots to vulnerable populations’ [18] (p. 4). Evidence already details shedding of antibiotic-resistant *Escherichia coli* by horses [18] (p. 2). Add to this poor waste management, an additional risk factor in AMR dispersal, and the importance of the migratory aspect of working equines becomes clear when considering AMR dissemination and the possibility that they could ‘act as efficient AMR dispersers’ [18] (p. 1) as they move between locations.

With a population of over half a million donkeys and mules in the country [19], India is home not only to some of the largest populations of donkeys and mules globally, but also to an already existent multidrug resistance (MDR, resistance to three or more drugs) amongst its equine population [20]. India was also reported to be the largest consumer of antibiotics globally in 2010, and has contributed collectively to 76 per cent of the increase in antibiotic usage between 2000 and 2010 alongside Brazil, Russia, China, and South Africa (BRICS) [21]. As a country, it is reported to have ‘some of the highest antibiotic resistance rates among bacteria that commonly cause infections in the community and healthcare facilities’ [22] (p. 2). AMR in India can result from a proliferation of ‘substandard, falsified, or counterfeit drugs’ [23] (p. 189) within the market, however, poor overall stewardship remains the primary cause [24].

Efforts by the government of India to control poor stewardship, especially in relation to community pharmacies, have been in place since the introduction of Schedule H listings of the Indian Drugs and Cosmetics Act 1940, which restricts the sale of certain drugs without the presence of a prescription. A general failure by drug retail outlet workers to comply necessitated, for some drugs, the introduction of Schedule H1 in 2014 [9], which continues to mandate the requirement of a prescription supplied by a registered veterinary practitioner, but also obliges greater levels of record keeping and data management. Pharmacy licences have been cancelled for non-compliance with the legislation, since this recent policy introduction, but few studies have been conducted since 2014 to evidence the impact of the new schedule [3].

This study uses a mixed-methods approach to examine the capacities of drug retail outlet workers and seeks to understand how said capacities, or lack of, might affect their antibiotic dispensing practices, particularly in relation to donkeys and mules.

## 2. Results

### 2.1. Respondent Characteristics

A total of 23 drug retail outlet workers were interviewed across all study sites. All respondents were male, with 61 per cent falling in the 30 to 50-age bracket, 22 per cent in the 18 to 30 bracket, and 17 per cent over the age of 50. All drug retail outlets, or medical stores as they are known locally, stocked both human and animal medicine apart from one, which specialised in animal medicine only. All SSI respondents were working during the interview process, except for one. This allowed for participant observation to occur concurrently with the interview by at least one researcher and one research assistant.

### 2.2. Training

Interview data revealed that 52 per cent of Drug Retail Outlet (DRO) workers reported having no formal training in pharmaceuticals at all. Moreover, 30 per cent reported possessing a D Pharm (Diploma in Pharmacy), 13 per cent claimed they had completed a B Pharm (Bachelor of Pharmacy), and only one respondent stated that he had an M Pharm (Masters of Pharmacy) (See Table 1). Furthermore, 13 per cent were in possession of a non-pharmaceutical related qualification, such as business and commerce, and 26 per cent admitted a 12th grade (16–18 years old, secondary) education only.

A total of 78 per cent of DRO workers reported having no formal training in animal-related pharmaceuticals, despite selling veterinary drugs.

### 2.3. Antibiotic Dispensing Practices

A total of 35 per cent of all DRO workers in the study sold antibiotics without a prescription, according to SSIs and patient simulations. Moreover, 57 per cent of those interviewed sold antibiotics (either overtly or covertly) without a prescription during the interview process or openly admitted to doing so, while only 18 per cent did so during patient simulations. The reason for this disparity might result from the fast ‘gossip networks’ existing within small communities and unknowing participants having been alerted to our presence. Patient simulations were always carried out post-interviews. Additional to this, the majority of medical stores selected for interview were identified by our key informants as being known suppliers to equine owners.

All DRO workers who sold antibiotics via both the patient simulation method and during the semi-structured interview process did so unprompted and without being specifically asked for antibiotics. The patient simulation sample could not be screened for specific customer-species type. Participant observation revealed that even some of the SSI respondents who claimed to never sell antibiotics without a prescription, did so whilst serving customers during interviews.

Antibiotics dispensed illegally included, in order of most common, amoxicillin, trimethoprim, tazobactam, tetracycline, norfloxacin, ampicillin, dicloxacillin, cloxacillin, ciprofloxacin, and ofloxacin.

Results from the SSIs and patient simulations were corroborated by focus group participants whose veterinary experience with equines in each region qualified them to comment on the dispensing practices of drug retail outlet vendors in their area. Members of both focus groups admitted that antibiotics are dispensed frequently without the DRO vendors requesting a prescription.

A qualified vet practitioner from Focus Group Discussion (FGD) 1 stated that even if a DRO vendor is aware of the correct dosage, they often dispense inappropriately for economic purpose.

“If the owner of the shop is intelligent enough and is wise enough, he knows what dosage is to be given, but multiple times, because of greediness, they would sell more than what is necessary”.

(Focus group respondent)

“Legally its banned, nobody can give anything without a prescription, but yes, goodwill relations, the greed of selling medicine, yeah, many things which goes under current”.

(Focus group respondent)

A qualified vet practitioner in FGD 2 stated that at least 40 per cent of drug retail outlets sell medicines without prescription.

Another focus group participant estimated that only 10 per cent of DRO vendors know anything about equine medicine. He stated that of those who have any veterinary-related knowledge, the majority know about production animals only, concluding that their dosing knowledge or diagnosing ability in relation to equines is ‘highly questionable’. Another FGD participant reported that, in their experience, many DRO vendors use a ‘trial and error’ method until equine illnesses reach a ‘terminal stage’ at which point they—the qualified veterinarians—are called in to intervene. Reportedly, owners with limited knowledge of how medication works tend to expect immediate results, and if their animal is not cured rapidly, they might reach out to a different DRO vendor who, with similarly limited knowledge, will prescribe a different antibiotic to the first. A FGD participant illustrates this point.

“There was an animal that was having respiratory distress. [The owner] went to the hospital and the hospital was closed. He went to the pharmacist, and the pharmacist, without knowing anything, he started an antibiotic and that is Ceftriaxone, a third generation antibiotic […] Second day. Because the animal was not ok, this person reaches out to a local practitioner and second person comes in and he gives […] Cloxicillin, which is a drug much lower than Ceftriaxone. So, two days already two sorts of antibiotic given. Second day evening, this man again calls me. The animal is still not ok, what should I do. So then I went through the history of drugs given […] and I saw different two days, two types of antibiotics given. I was stuck. Ideally in that case, I would never have gone for antibiotic”.

(Focus group respondent)

Of the antibiotics dispensed, only 21 per cent were correctly dispensed for the symptoms presented, but all dosages were under-dosed and therefore incorrect. The only interview respondent to dispense the correct antibiotic (but at the incorrect dosage) for any of the symptoms presented, did not possess a pharmaceutical-related qualification.

Of the 23 DRO vendors interviewed, 43 per cent of DRO workers incorrectly believe that some antibiotics can be legally dispensed without a prescription, while others were aware of the legalities of antibiotic dispensing, several excused their illegal dispensing behaviours due to the inaccessibility of veterinary practitioners in their locality.

“In this region there is no VS, a veterinary surgeon, so there is nobody who can write a prescription, so they come to me”.

(DRO vendor)

### 2.4. Anecdotal Reports of AMR

Awareness of AMR was greater amongst FGD respondents, although some drug retail outlet vendors also demonstrated awareness of the existence of AMR as a global issue and that poor prescribing practices played a significant role in its development. Several respondents referred anecdotally to personal experience or hearsay of the development of AMR within their localities:

“[He has] seen cases of antimicrobial resistance and this is the practice of under-dosing of antibiotics by the medical stores. [He is] saying that animals have grown resistance to Oxytetracycline and Penicillin. It does not work anymore”.

(DRO vendor)

*“[Which antibiotics do they know there is resistance to now?] Enrofloxacin, Oxytetracycline”*.

(Focus group respondent)

A FGD respondent also cited anecdotal evidence of antimicrobial resistance occurring in equines within the region within which he works, which he attributes to an ‘indiscriminate use of antibiotics’. He also stated that greater doses of antibiotics are now required for infections where years prior, a lower dosage was required.

## 3. Discussion

This is the first study examining antibiotic stewardship of drug retail outlet vendors in northern India in relation to working equids. It aimed to examine the dispensing practices of drug retail outlet workers in northern India in terms of donkey and mule disease, in order to identify their role as social drivers in the development of antimicrobial resistance in animals. Understanding species-specific dispensing practices is essential in the identification of training and skills gaps, which might be bridged in order to improve antimicrobial stewardship in areas where pharmacies are likely be the primary service provider for animal owners.

Despite the introduction of the Schedule H1 notification in India in 2014, which stipulates that drugs falling within this classification (including all antibiotics) can only be dispensed against a valid prescription, this study has revealed that equine owners in northern India are frequently being sold antibiotics without the need for a prescription and, in most cases, with incorrect diagnoses and insufficient dosage. This study complements similar findings in Peru [7], Sri Lanka [25], Spain [26], and India [6] where pharmacists have been proven to lack the necessary stewardship practices required to ensure responsible antimicrobial usage, by either not being adequately trained or qualified, or by engaging in inappropriate dispensing practices in spite of this. According to Bebell and Muiru [1], ‘antibiotics are misused in all regions of the world’ [1] (p. 350). The originality of this research lies in evidencing prescribing practices of drug retail outlet vendors, not only for animals, but also for a specific genus.

The prevalence of unqualified staff dispensing medicine to animal owners is widespread across the three study regions of northern India, signifying that enforcement of regulation is light, and government inspection is either failing to identify, or is overlooking, drug retail outlets where the licensing arrangement is lax. Our findings also demonstrate the limited proportion of animal-related training amongst all respondents, whether formally or informally trained, further compounding the knowledge gap of all drug retail outlet vendors. The global pattern of over-the-counter and inappropriate sales of antibiotics demonstrates that ‘the ratio of use in animals as compared to humans is astounding’ [27] (p. 3). Further investigation is therefore required into the role of drug retail outlets in the development of antimicrobial resistance among animals. Lack of drug retail outlet vendor knowledge of equines in comparison to their knowledge and experience with higher population species means that dispensing practices in terms of donkeys and mules is likely to be poorer than for most other large animals, putting equines at greater risk of antibiotic resistance in the future.

Such high frequencies of unqualified staff threaten to increase antimicrobial resistance in all three study areas, a danger intensified by the mobile nature of working equids. Donkey and mule owners also belong to some of the poorest sections of society in India, an additional concern as it has been reported that ‘shedding in faeces was significantly higher in equids maintained by low-income people’ [20] (p. 266). A vicious cycle is therefore probable. Antimicrobial resistance is likely to occur at a higher frequency within communities where poor prescribing practices are prevalent. These communities tend to be located in more rural areas, which often means high levels of poverty; thus, already vulnerable populations are ultimately made more vulnerable by this cycle.

Absence of training is, however, not the only driver for poor antimicrobial stewardship amongst drug retail outlet workers. Our findings demonstrate a significant pattern of poor prescribing and dispensing by those who declared themselves as qualified. This suggests a failure at the education level for all curriculums; D Pharm, B Pharm, and M Pharm, as all respondents citing formal training as a personal capacity dispensed antibiotics incorrectly according to symptoms. Interestingly, the only vendor to dispense the correct antibiotic, although at an incorrect dosage, did not describe himself as formally trained. This signifies that where training does exist, it is severely lacking, especially in relation to equine disease and treatment. Barker et al., [6] attributes this failure partly to ‘a knowledge gap of over two decades’ [6] (p. 5) as national standards to the curriculums were last updated in 1991. The quality of training at institutions as well as the lack of obligation to partake in continuing education in pharmaceuticals is also blamed for DRO vendors’ lack of knowledge regarding antibiotics and resistance [6].

Previous studies of pharmacies in low- and middle-income countries suggest that community pharmacists engage in inappropriate dispensing of antibiotics for a variety of reasons. Firstly, the desire to make a sale might take precedence over safe and appropriate dispensing of antibiotics [25], a factor suggested by several focus group participants. Secondly, under-dosing caused by shortened courses of antibiotics being sold, is often considered an appropriately ‘charitable’ response to poorer customers’ requests [28].

A similar issue was described by respondents in our study:

“If I write an antibiotic for three to five days they can’t even afford for a single day, forget five days”.

(DRO Vendor)

Thirdly, poor stewardship is attributed to lack of professionally trained pharmacists [29], although in the case of northern India, this does not appear to impact upon knowledge of antimicrobial resistance and related social drivers [6].

Where some categories of customer have been known to ask specifically for antibiotics in a self-treatment approach, cited as a form of pressure by pharmacists in other studies to supply what the consumer demands [7], this is unlikely in the case of mule and donkey owners due to limited pharmaceutical knowledge. The onus, therefore, lies more on the prescribing practices of the medical store vendors in this instance, rather than the customer.

It is clear that antibiotics are still easily available within the regions of study; however, our figures suggest that the introduction of the H1 scheduling might have positively impacted the dispensing practices of community pharmacies. Jaganathan et al. [9] carried out a study prior to the introduction of the H1 schedule and over 70 per cent of store vendors were willing to dispense antibiotics without prescription. Our findings, although stemming from a different region, and using animals as patients rather than humans, demonstrate a lower incidence of illegal antibiotic sales.

The combination of insufficient training, illegal registration and licensing ensuring qualified pharmacists are rarely, if ever, on the premises, and subsequent limits in knowledge regarding equines, drivers of antimicrobial resistance, and the appropriate and legal use of antibiotics themselves, all threaten the future of effective disease management of equine populations in India. Poor patient outcome, owner livelihood, and resistance patterns constitute ‘negative consequences at the individual and societal level’ [6] (p. 2). Anecdotal reports of antimicrobial resistance amongst the equine population of northern India remain unconfirmed, but should be considered in further research.

## 4. Methods

This study examined the antibiotic dispensing practices of fifty-one medical store workers and the vendor capabilities of twenty-three of those workers in northern India using a mixed-methods approach, the combination of which offers new, in-depth insights into the antimicrobial stewardship practices of medical store owners in northern India. The depth of the qualitative data accrued from the sample size, and the ability to triangulate resulting data from all methods employed, was sufficient to reach data saturation.

Fieldwork took place from February to March 2019 in three regions of northern India: Faridabad, Haryana; Neemrana, Rajasthan; and Lucknow, Uttar Pradesh. As one of the largest consumers of antibiotics in the world [3], and home to a high population of mules and donkeys, two species largely ignored within this research agenda, India provides a suitable area of study to examine vendor capacities and practices. The significant number of brick kilns and construction sites known to use working equines in the region led to the selection of northern India for the study [30]. In addition, the presence of a local partner (Donkey Sanctuary India (DSI)) allowed for the provision of logistical support where required as well as assistance in identifying appropriate study locations using key informants.

Within these regions, areas of study consisted of rural, peri-urban and urban areas. The selected areas were chosen due to their known populations of brick kilns and construction sites where donkey and mule owners were prevalent, and key informants belonging to a variety of non-governmental organisations acted as gatekeepers in order to enable access to the drug retail outlets. The research formed part of a wider study examining the role of drug retail outlets on equine welfare in India. The number of DROs studied, therefore, pertains to the number available and open at the time selected sites were visited.

### 4.1. Semi-Structured Interviews (SSIs)

Respondents were recruited across the three regions through purposive sampling. Their relevance to the sample was based upon whether donkey or mule owners were represented within their client base.

The semi-structured interviews explored the capacity and capability-building history of the vendors who were on-site at the time of the interview, principally through probes regarding qualifications and training. Interviews lasted 15 to 73 min, with an average interview length of 31 min.

Consent was obtained verbally, audio recorded and stored securely to comply with data protection.

### 4.2. Vignettes

Dispensing practices were assessed through the incorporation of theoretical equine-patient vignettes into the semi-structured interviews. Respondents were requested to outline how they would manage a particular case study of a donkey or mule with typical histories and symptoms presented in a verbal format where time and respondent compliance allowed. The principal cases presented were respiratory disease, lameness, colic and fever.

Both the semi-structured interview and the vignette knowledge assessment were conducted by English-speaking researchers and translated in-situ using Hindi-speaking Indian nationals. They were audio-recorded in-situ and transcribed post-fieldwork.

### 4.3. Patient Simulations

Patient simulations using mock equine owners were carried out to further enable the assessment of antibiotic dispensing practices of vendors in those areas. Moreover, 28 drug retail outlet vendors’ dispensing practices were recorded through the use of patient simulation. Simulations were carried out in the same districts as the interviews, but participants were distinct from those interviewed and selected purposively, according to their location in relation to both donkey and mule owning communities, as well as their proximity to the other pharmacy vendors partaking in the semi-structured interviews. Two Indian national research assistants were trained and advised on ‘acting’ the part of a donkey or mule owner and each visited stores separately and anonymously, outlining simple symptoms of lameness or respiratory disease to the vendors. Immediately upon leaving the store, the research assistants inputted all information arising from the interaction into an Open Data Kit (ODK) Collect pre-designed form on digital tablets, commenting on what medicines were dispensed, including dosage and treatment length, whether the vendor requested further information regarding the condition, and whether advisory information such as side effects, contraindications, or non-pharmaceutical advice was provided without prompting. All information was uploaded to a central server. All patient simulations were conducted entirely in Hindi, and results were recorded in English for interpretation and analysis.

Few equine owners were reported, during interviews, as specifically asking for antibiotics and this was therefore avoided in the patient simulation exercise, in order to avoid suspicion.

Subjects of the patient simulation were not informed post-fieldwork of their participation in the research but no specific records were maintained which could identify either outlet or vendor.

### 4.4. Focus Groups

Focus group discussions were run with a purposive sample of animal health service practitioners outside of DROs, such as veterinarians, para-veterinarians, and community animal health workers (CAHWs). Two different group discussions were held, made up of five and six participants respectively. Each focus group lasted approximately 65 min and involved open-ended questions regarding the respondents’ knowledge, experience, and perceptions of the role of veterinary drug retail outlets on equine welfare in the aforementioned areas. Focus groups were audio-recorded, translated in-situ, and later transcribed.

### 4.5. Participant Observation

Aside from participant observation carried out actively during the patient simulations, researchers and research assistants performed participant observation throughout the semi-structured interview process, ascertaining where antibiotics were sold without prescription and whether prompted or unprompted, while DRO workers were working. For the assessment of antibiotic dispensing practices, it was difficult to ascertain whether a prescription had been provided in some cases; for example, where the researcher observed a note being handed to the DRO, which did not appear official. To provide the most conservative estimate of dispensing practices that occurred without a prescription, these uncertainties were classed as ‘with prescription’.

### 4.6. Analysis

All interview and focus group transcripts were uploaded to, and analysed, using qualitative software package Nvivo (V.12.2, QSR International). Both inductive and deductive coding arose from the data, resulting in themes pertaining to the original research aims. These themes were then reviewed by the corresponding researcher for agreement on findings and outcomes. Vendor responses to vignettes and patient simulations were assessed by two registered veterinary practitioners.

### 4.7. Using a Mixed-Methods Approach

A mixed-methods approach was adopted to strengthen the credibility of the study. By cross-checking ‘multiple accounts of social reality’ [31] (p. 377) using semi-structured interviews, patient simulation, focus groups and participant observation, efficient triangulation of data is enabled. The self-reporting of drug retail outlet vendors, which had the potential for bias, with evidence of actual dispensing behaviours of similar vendors in the locality, cross-checked with the knowledge and experience of focus group participants, and our own observations during interviews, supports ‘greater confidence in findings’ [31] (p. 379).

The research was carried out under the research policy and guidelines of The Donkey Sanctuary and received approval from the executive team therein. All participants provided informed consent to participate.

## 5. Conclusions

Pharmaceutical legislation in India decrees that all principal vendors working in medical stores are suitably qualified and licensed to dispense drugs. It also mandates, as a legal requirement, presentation of a medical prescription for all Schedule ‘H’ and ‘H1′ drugs, which covers all antibiotics, prior to their dispensing. However, our study revealed a clear pattern of this legislation being ignored in vendor practices and behaviour. This is likely to lead not only to the poor welfare of equines, but also to an increase in antimicrobial resistance amongst equine populations in northern India and, due to the mobile nature of donkeys and mules, neighbouring regions and countries.

Although DRO workers are ‘primary antibiotic gatekeepers’ [6] (p. 7) with a significant stewardship role, these findings illustrate that a substantial gap in capacities exists amongst drug retail outlet workers in northern India, with few being either sufficiently qualified or trained to dispense antibiotics to humans, much less animal owners. This is especially the case in relation to equine disease. The study highlights the need for further training of private DRO workers as well as knowledge extension and awareness training for both DRO workers and animal owners regarding antimicrobial resistance and its potential impact upon livelihoods. It also stresses the need for greater enforcement of regulation at all levels.

Simultaneously, with greater enforcement of over-the-counter dispensing regulations, it should also be recognised that in rural areas where veterinary hospitals or practitioners may be scarce, this may negatively impact upon equine owners’ accessibility to medicine, and consequently upon their livelihoods. Further studies are therefore required to identify realistic interventions to bridge what has become a ‘wicked problem’ [32] in India and other less economically developed countries.

## Figures and Tables

**Table 1:** Results outlining levels of training and antibiotic dispensing practices (semi-structured interview (SSI) n = 23, patient simulation n = 28). DRO = Drug Retail Outlet.

Question/Observation	Response		Percentage *	No.	Method
**Training**					
What level of education (pharmaceutical or otherwise) do you have?	Not trained	Non-related education	52	12	SSI
	Trained	D pharm	30	7	SSI
		B pharm	13	3	
		M pharm	4	1	
Do you possess animal-specific pharmaceutical training?	No		78	18	SSI
	Yes		13	3	
	No response		9	2	
**Antibiotic Dispensing Practices**					
DRO vendors that sold antibiotics	Without prescription		35	18	SSI and Patient simulation
	With prescription/none sold		65	33	
	Without prescription		57	13	SSI
	With prescription/none sold		43	10	
	Without prescription		18	5	Patient simulation
	With prescription/none sold		82	23	
Can some antibiotics be legally dispenses without prescription?	Yes		43	10	SSI
	No		57	13	

* Percentages may not add up to 100% due to rounding.

## Data Availability

The datasets generated and/or analysed during the current study are not publicly available due to them containing sensitive material, which do not meet the GDPR guidelines.

## References

[1] Bebell L.M., Muiru A.N. (2014). Antibiotic Use and Emerging Resistance: How Can Resource-Limited Countries Turn the Tide?. Glob. Hear..

[2] Fishman N. (2006). Antimicrobial stewardship. Am. J. Infect. Control.

[3] Laxminarayan R., Chaudhury R.R. (2016). Antibiotic Resistance in India: Drivers and Opportunities for Action. PLoS Med..

[4] Holmes A., Moore L., Sundsfjord A., Steinbakk M., Regmi S., Karkey A., Guerin P.J., Piddock L.J.V. (2016). Understanding the mechanisms and drivers of antimicrobial resistance. Lancet.

[5] Dyar O.J., Huttner B., Schouten J., Pulcini C. (2017). What is antimicrobial stewardship?. Clin. Microbiol. Infect..

[6] Barker A.K., Brown K., Ahsan M., Sengupta S., Safdar N. (2017). What drives inappropriate antibiotic dispensing? A mixed-methods study of pharmacy employee perspectives in Haryana, India. BMJ Open.

[7] Redding L.E., Barg F.K., Smith G., Galligan D.T., Levy M.Z., Hennessy S. (2013). The role of veterinarians and feed-store vendors in the prescription and use of antibiotics on small dairy farms in rural Peru. J. Dairy Sci..

[8] Auta A., Hadi M.A., Oga E., Adewuyi E.O., Abdu-Aguye S., Adeloye D., Strickland-Hodge B., Morgan D.J. (2019). Global access to antibiotics without prescription in community pharmacies: A systematic review and meta-analysis. J. Infect..

[9] Jaganathan M., Bn V., Mayathevar B., Mahato R. (2016). Non-prescription sale of antibiotics in pharmacies across Puducherry, India. Int. J. Basic Clin. Pharmacol..

[10] Shet A., Sunderesan S., Forsberg B. (2015). Pharmacy-based dispensing of antimicrobial agents without prescription in India: Appropriateness and cost burden in the private sector. Antimicrob. Resist. Infect. Control.

[11] Higham L.E., Ongeri W., Asena K., Thrusfield M.V. (2016). Characterising and comparing drug-dispensing practices at animal health outlets in the Rift Valley, Kenya: An exploratory analysis (part II). Trop. Anim. Health Prod..

[12] Chauhan A.S., George M.S., Chatterjee P., Lindahl J.F., Grace D., Kakkar M. (2018). The social biography of antibiotic use in smallholder dairy farms in India. Antimicrob. Resist. Infect. Control..

[13] Barrandeguy M., Carossino M. (2018). Infectious Diseases in Donkeys and Mules: An Overview and Update. J. Equine Vet. Sci..

[14] McNeill W.H. (1987). The eccentricity of wheels, or Eurasian transportation in historical perspective. Am. Hist. Rev..

[15] Höger A., Mayo M., Price E., Theobald V., Harrington G., Machunter B., Low Choy J., Currie B., Kaestl M. (2016). The melioidosis agent Burholderia pseudomallei and related opportunistic pathogens detected in faecal matter of wildlife and livestock in northern Australia. Epidemiol. Infect..

[16] Von Wintersdorff C., Penders J., Van Niekerk J.M., Mills N.D., Majumder S., Van Alphen L.B., Savelkoul P.H.M., Wolffs P. (2016). Dissemination of Antimicrobial Resistance in Microbial Ecosystems through Horizontal Gene Transfer. Front. Microbiol..

[17] Pearson R.A., Krecek R.C. (2006). Delivery of health and husbandry improvements to working animals in Africa. Trop. Anim. Health Prod..

[18] Arnold K.E., Williams N., Bennett M. (2016). Disperse abroad in the land’: The role of wildlife in the dissemination of antimicrobial resistance. Boil. Lett..

[19] DAHDF (2014). 19th Livestock Census—2012: All India Report.

[20] Singh B., Babu N., Jyoti J., Shankar H., Vijo T., Agrawal R., Chandra M., Kumar D., Teewari A. (2007). Prevalence of Multi-Drug-Resistant Salmonella in Equids Maintained by Low Income Individuals and on Designated Equine Farms in India. J. Equine Vet. Sci..

[21] Van Boeckel T.P., Gandra S., Ashok A., Caudron Q., Grenfell B.T., A Levin S., Laxminarayan R. (2014). Global antibiotic consumption 2000 to 2010: An analysis of national pharmaceutical sales data. Lancet Infect. Dis..

[22] Gandra S., Joshi J., Trett A., Lamkang A.S., Laxminarayan R. (2017). Scoping Report on Antimicrobial Resistance in India.

[23] Mendelson M., Røttingen J.-A., Gopinathan U., Hamer D.H., Wertheim H., Basnyat B., Butler C., Tomson G., Balasegaram M. (2016). Maximising access to achieve appropriate human antimicrobial use in low-income and middle-income countries. Lancet.

[24] Torres N.F., Chibi B., Middleton L.E., Solomon V.P., Mashamba-Thompson T.P. (2018). Evidence of factors influencing self-medication with antibiotics in LMICs: A systematic scoping review protocol. Syst. Rev..

[25] Zawahir S., Lekamwasam S., Aslani P. (2019). Antibiotic dispensing practice in community pharmacies: A simulated client study. Res. Soc. Adm. Pharm..

[26] Zapata-Cachafeiro M., Gonzalez-Gonzalez C., Vazquez-Lago J.M., López-Vázquez P.M., López-Durán A., Smyth E., Figueiras A. (2014). Determinants of antibiotic dispensing without a medical prescription: A cross-sectional study in the north of Spain. J. Antimicrob. Chemother..

[27] Builder M. (2014). Antimicrobial Resistance as an Emerging Threat to National Security.

[28] Barker A.K., Brown K., Ahsan M., Sengupta S., Safdar N. (2017). Social determinants of antibiotic misuse: A qualitative study of community members in Haryana, India. BMC Public Health.

[29] Sakeena M., Bennett A., McLachlan A. (2018). Non-prescription sales of antimicrobial agents at community pharmacies in developing countries: A systematic review. Int. J. Antimicrob. Agents.

[30] Watson T.L., Kubasiewicz L.M., Chamberlain N., Nye C., Raw Z., Burden F.A. (2020). Cultural “Blind Spots,” Social Influence and the Welfare of Working Donkeys in Brick Kilns in Northern India. Front. Vet. Sci..

[31] Bryman A., Bell E. (2007). Social Research Methods.

[32] Beerlage-de Jong N., Van Gemert-Pijnen L., Wentzel J., Hendrix R., Siemons L. (2017). Technology to support integrated antimicrobial stewardship programs: A user centered and stakeholder driven development approach. Infect. Dis. Rep..

